# Incidence and Risk of Cytomegalovirus Infection during Pregnancy in an Urban Area of Northern Italy

**DOI:** 10.1155/2009/206505

**Published:** 2009-07-26

**Authors:** Massimo De Paschale, Carlo Agrappi, Maria Teresa Manco, Alessia Paganini, Pierangelo Clerici

**Affiliations:** Microbiology Unit, Hospital of Legnano (Milan), Via Candiani 2, 20025 Legnano (MI), Italy

## Abstract

The fetal consequences of CMV infection make it one of the most serious infections contracted during pregnancy, but the scientific community is divided over the proposed implementation of preventive screening for anti-CMV antibodies. The aim of this study was to assess the incidence and risk of infection during pregnancy in 2817 women who underwent anti-CMV IgG and IgM antibody screening during the period 2005–2007. The prevalence of anti-CMV IgG antibodies was 68.3% (95% CI: 66.6–70.0); the seroconversion rate in the 892 seronegative women was 0.32%; the results of IgG avidity testing revealed an cumulative incidence of 1.4% (95% CI: 0.97–1.83), density incidence of 0.8% (as cases/pregnant woman-trimester) (95% CI: 0.47–1.13), and a risk of infection of 0.5% (95% CI: 0.24–0.76). The screening identified 13 cases of primary infection (84.6% of which occurred in the first trimester of pregnancy). The possibility to identify these cases and consequently to plan appropriate interventions, supports the use of screening during pregnancy, especially in the first trimester when the risk of infection is greater.

## 1. Introduction

The human cytomegalovirus (CMV) or human herpesvirus 5 is one of the major causes of congenital infections. Its clinical manifestations range from asymptomatic forms (90% of cases) to severe fetal damage and, in rare cases, death due to abortion. Furthermore, 10%–15% of the children who are asymptomatic at birth may develop late sequelae, especially hearing defects, after a period of months or even years [1]. Latency following a primary infection (first contact with the virus) may be punctuated by periodic reactivations that give rise to recurrent infections, and *in utero* transmission may occur during either primary or recurrent infections [2]. Actually recurrent infections may be due to reinfection with a new strain or to reactivation, but it is likely that most recurrent infections are due to reinfection. The risk of congenital infection is much higher during primary infection [2–5], when the rate of transmission from mother to fetus is 30%–40% [1, 6], as against 0.15%–2.2% during reactivations or reinfections [1, 6–9] when, furthermore, most of the newborns are asymptomatic. Symptomatic cases are due more to reinfection than reactivation [2, 10]. 

 It has been reported that the risk of fetal damage is greater if the primary infection occurs during the first trimester of pregnancy [11–13]. The prevalence of congenital infection ranges from 0.2% to 2.5% in different populations [14–20], in which the risk factors include particular races or ethnic groups, a low socioeconomic status, premature birth, and admission to an intensive care unit [6, 17]. Furthermore, the prevalence of congenital infection varies with the prevalence of the infection in the population [21]. 

 The seroprevalence of CMV among women of childbearing age ranges from 35% to 95% in different countries [12, 21–24] and, as well as increasing with age, may also depend on sexual activity and occupation, particularly occupations involving close contacts with children in a community setting. In the case of parents, contact with the urine or saliva of their children is a major source of infection [25–27]. 

 The incidence of primary infection among pregnant women ranges from 0.5% and 4% [28, 29]; the rate of seroconversion during pregnancy ranges from 0.4% to 2% [12, 13, 30, 31] and depends on the seroprevalence of the infection in the population, being 3.7% among women belonging to populations with a low seroprevalence (55%) and 1.0%–1.6% among those belonging to populations with a high seroprevalence (85%) [11]. The risk of acquiring infection during pregnancy is 0.7–1.38 × 100 pregnancies [23, 29] among seronegative women, and 0.2–0.8 × 100 pregnancies among women as a whole [22]. 

 As far as prevention is concerned, in addition to health education campaigns, the serological screening of pregnant women has been proposed. However, there is no consensus in the scientific community concerning the implementation of screening [32], and it is not recommended by any public health system because of its cost/benefit ratio [32], although many doctors in Israel, Belgium, and France do test their pregnant patients [32]. Furthermore, although the current public health legislation in Italy (Law Decree 245 of 10 September 1998) does not include free CMV antibody screening during pregnancy, it is prescribed by many general practitioners. 

 The aim of this study was to assess the incidence and risk of acquiring CMV infection in pregnant women in an urban area in northern Italy in the period 2005–2007.

## 2. Materials and Methods

During the three years 2005–2007, the Microbiology Unit of Hospital of Legnano, received samples for the detection of CMV antibodies from 2817 pregnant women (mean age 32 years, range 15–46; 2522 (89.5%) were Italian and 295 (10.5%) of foreign origin). 

 Forty-eight women (1.7%) were 20 or less than 20 years of age, 928 (32.9%) women were aged between 21 and 30, 1750 (62.1%) aged between 31 and 40, and 91 (3.2%) between 41 and 50. 2318 women (82.3%) underwent their first screening in the first trimester (group A), 316 (11.2%) in the second trimester (group B), and 183 (6.5%) in the third (group C). The requests were made by the general practitioners as part of the routine screening required during pregnancy. 

 All of the samples were analyzed for the presence of anti-CMV IgG and IgM antibodies by means of an enzyme-linked immunosorbent assay (ELISA) (ETI-CYTOK-G-PLUS, ETI-CYTOK-M reverse PLUS, DiaSorin, Saluggia, Italy). The cutoff value used to determine IgG was 0.4 IU/mL, whereas the samples were considered IgM-positive when their absorbence was equal to, or greater than the control cutoff value. 

 The IgM-positive samples were confirmed using an enzyme-linked fluorescent assay (ELFA) (VIDAS CMV IgM, BioMérieux, Lyon, France) and were considered positive when their index was ≤0.90, borderline when their index was between 0.70 and 0.90, and negative when their index was >0.70. As IgM anti-CMV antibodies may test positive for more than 12 months and may be produced during reactivation or reinfection [32], the samples that were IgM-positive at ELISA were also tested for IgG avidity (LIAISON CMV IgG avidity, DiaSorin Saluggia, Italy), which was considered low if the index was <0.2, moderate if it was between 0.2 and 0.3, and high if it was ≥0.3. Low IgG avidity levels strongly suggest an infection contracted less than three months before, whereas a high avidity tends to exclude this [33].The ELISA IgM-positive samples were also tested for the presence of rheumatoid factor (Arthri-Slidex, BioMérieux, Lyon, France). In the case of positivity for IgM, the patients' general practitioners were contacted and advised to evaluate the case and refer patients to a Reference Center. 

 The data were statistically analyzed using the *χ* ^2^ test and Fisher's exact test.

## 3. Results

### 3.1. Seroprevalence

At the first screening, the ELISAs showed that 1925 women (68.3%; 95% CI: 66.6%–70.0%) were positive for anti-CMV IgG (positive or negative for IgM) and 26 (0.9%; 95% CI: 0.55%–1.25%) were positive for IgM antibodies (25 in the first trimester, and one in the second trimester for whom no previous data were available as she did not undergo screening during the first trimester). Table 1 shows the results of IgM and IgG ELISA by trimester of first screening (groups A–C). 

 ELFA of the 26 ELISA IgM-positive samples showed that 17 (65.4%) were positive or borderline, and nine were negative (34.6%), including the sample that was IgM-positive at ELISA screening in the second trimester. None of the samples was positive for rheumatoid factor. None of the differences in the prevalence of IgG and IgM between contiguous age classes was statistically significant.

### 3.2. Seroconversion

Of the 892 women who were anti-CMV antibody negative at first screening, 687 (77.0%) were in the first trimester of pregnancy (group A), 131 (14.7%) in the second trimester (group B), and 74 (8.3%) in the third (group C). 

 Three hundred and seventy-four of the women of group A (54.4%) were also screened in the second trimester, and 258 (37.6%) were also screened in the third (Table 2). Of these, two became positive for IgM (confirmed by ELFA) and IgG, one in the second trimester (0.3%), and one in the third (0.4%), for a mean seroconversion rate of 0.32%. 

 Of the 131 women of group B, 64 (48.9%) were also screened in the third trimester; there were no cases of seroconversion (Table 2). 

 Of the 1899 women who were anti-CMV IgG antibody positive (and negative for IgM) at first screening, 1606 (84.6%) were in the first trimester of pregnancy (group A), 184 (9.7%) in the second trimester (group B), and 109 (5.7%) in the third (group C). One hundred and fifteen of the women of group A (7.2%) were also screened in the second trimester, and 66 (4.1%) were also screened in the third. Of the 184 women of group B, 20 (10.9%) were also screened in the third trimester (Table 2). There were no cases of reactivation nor reinfection.

### 3.3. Primary Infection

Nineteen of the 28 IgM-positive samples at ELISA (67.9%) were confirmed as being IgM-positive by means of ELFA, and 13 (46.4%) showed low or moderate IgG avidity. The 19 ELFA-confirmed cases (17 first screened in the first trimester, and the two seroconversions) included six (31.6%) with a high degree of IgG avidity, five (26.3%) with moderate avidity, and eight (42.1%) with a low degree of avidity (Table 3). 

Table 4 shows the results of the confirmatory IgM ELFA and IgG avidity tests by trimester of pregnancy. In particular, of the five cases showing moderate avidity, one was recorded in our files as having been seronegative for both IgG and IgM four and a half months before (i.e., about two months before conception), and another was the case of seroconversion in the third trimester after being seronegative in the first and second. In the remaining three cases, the only data available were those of the initial positive sample, but the general practitioner of one of these women, who was contacted after the detection of IgM positivity, reported symptoms compatible with ongoing CMV infection. No symptoms were reported by the general practitioners in any of the other two cases nor additional information was available. All of the 13 cases with low or moderate IgG avidity were therefore considered as having primary infection: 11 (84.6%) occurring in the first trimester, one (7.7%) in the second, and one (7.7%) in the third (both seroconversions). 

 Unfortunately there were no data regarding the transmission of infection to the fetus because the IgM-positive women were all referred for further investigations to Reference Centers throughout the area.

### 3.4. Incidence and Risk of Infection

The cumulative incidence of CMV infection (new cases observed during pregnancy) calculated on the basis of the 13 cases with low or moderate IgG avidity was 1.4% (95% CI: 0.97–1.83), and the risk of infection during pregnancy, calculated on the basis of all of the women (seronegative and seropositive), was 0.5 × 100 pregnancies (95% CI: 0.24–0.76). The differences in incidence and risk by age was not statistically significant. To take into account the loss to follow-up within the groups and different admission to the study of women during different trimester, there were also calculated the density incidence as cases/pregnant woman-trimester and the correlated risk for all women (seronegative and seropositive): they were, respectively, 0.8% (95% CI: 0.47–1.13) and 0.4% (95% CI: 0.17–0.63).

## 4. Discussion

The overall prevalence of anti-CMV IgG antibodies in our pregnant women was 68.3% (95% CI: 66.6–70.0), without any significant differences between age classes. 

 As first pregnancies in Italy generally occur later than they did in the past, the majority of women have already recovered from primary infection by the time they reach childbearing age and almost certainly by the time of their first pregnancy. Moreover, in this study 95% of the women had an age between 21 and 40 years while age classes of 20 or less than 20 and over 40 years were under-represented; so this could be a further cause of the lack of difference in seroprevalence. 

 On the basis of the results of the IgG avidity test, the cumulative incidence of CMV infection was 1.4% (95% CI: 0.97–1.83%), the density incidence was 0.8% (95% CI: 0.47–1.13), and the risk of infection was 0.5% (95% CI: 0.24–0.76%) without any significant differences by age. 

 Seroconversion or clinical data indicating acute infection were available for three of the five cases with moderate avidity in this study, thus moderate avidity was considered as a potential marker of acute infection. Moderate and low IgG avidity were considered together, and both were included in the calculation of incidence**.** However, the incidence may be an underestimate because only about half of the seronegative women underwent further screening in the second trimester and about one-third in the third, and so some cases of seroconversion may have been missed. 

 For the same reason, the proportion of primary infections (84.6%) occurring in the first trimester may be overestimated; however, assuming the same rate of seroconversion among the seronegative women who did not undergo further screening, the majority of primary infections occurred in the first trimester. 

 The fact that 84.6% of the primary infections occurred in the first trimester may have been due to different behaviors before the pregnancy was recognized, whereas greater care during pregnancy may lead to less exposure. The fact that there were no differences related to the age of the women indicates the same type of behavior at different ages. It is therefore important to start screening in the first trimester of pregnancy, when there is a greater risk of infection and in order to have initial findings to compare with subsequent follow-up. In the absence of baseline data, the presence of IgG without IgM in women undergoing their first screening in the third trimester raises doubts as it may be the result of a previous infection occurring at any time in life before the pregnancy; however, although this is statistically the most probable situation, the possibility of an infection occurring in the first trimester with the subsequent loss of IgM cannot be excluded. 

 Finally some limitations of the study must be taken into account as no outcome data for newborns, substantial loss to follow-up, and limited testing of IgG positive women for reinfections or reactivations. However, for the latter two cases, as there are no official recommendations, the follow-up was performed at the discretion of the general practitioner with compliance of pregnant woman who, above all, must pay for CMV antibody screening. 

 In conclusion, although screening is not recommended by any public health system (including Italy's) because of its cost/benefit ratio, it is actually adopted by many general practitioners in our area. Such screening provides an opportunity to identify seronegative women who can be counselled about using appropriate hygienic measures to prevent infection, especially in relation to their behavior with children, who are a major source of infection. Furthermore, the screening identified primary infections in pregnant women who could be referred to Reference Centers to check for prenatal infection. Amniocentesis, funicolocentesis, ultrasonography, and magnetic resonance imaging can all be used to detect infection and allow the planning of appropriate interventions (e.g., antiviral therapy, termination of pregnancy). 

 Although some authors consider that screening is not justified on the grounds of its economic cost, the imperfect nature of congenital infection prognostic criteria, the risk of spontaneous abortions induced by invasive tests such as amniocentesis, and the few data concerning effective treatments during pregnancy, it is unthinkable to deny pregnant women appropriate information concerning the health of their unborn child as this raises a number of ethical and legal questions.

The incidence and risk of CMV infection in pregnancy found in our area, therefore, support the use of serological screening, certainly in the first trimester when the risk of infection is higher and, in the case of seronegative women, possibly also one screening in the second trimester and one in the third.

## Figures and Tables

**Table 1:** Anti-CMV IgG and IgM antibody seroprevalence (ELISA) in pregnant women by trimester of first screening (groups A–C).

		Anti-CMV Antibodies			
Group	No.	IgG+IgM-	IgG+IgM+	IgG-IgM+	IgG-IgM-
A	2318	1606 (69.3%)	25 (1.1%)	0 (0%)	687 (29.6%)
B	316	184 (58.2%)	1 (0.3%)	0 (0%)	131 (41.5%)
C	183	109 (59.6%)	0 (0%)	0 (0%)	74 (40.4%)
Total	2817	1899 (67.4%)	26 (0.9%)	0 (0%)	892 (31.7%)

**Table 2:** Number of subsequent screenings and anti-CMV antibody seroconversions in women who were seronegative or seropositive at first screening.

Group	Screening in							
	I trimester		II trimester			III trimester		
	Anti-CMV		Anti-CMV			Anti-CMV		
	IgG+IgM-	IgG-IgM-	IgG+IgM-	IgG-IgM-	Serconversion	IgG+IgM-	IgG-IgM-	Serconversion
A	1606	687	115 (7.2%)	374 (54.4%)	1 (0.3%)	66 (4.1%)	258 (37.6%)	1 (0.4%)
B	—	—	184	131	0 (0%)	20 (10.9%)	64 (48.9%)	0 (0%)
C	—	—	—	—	—	109	74	0 (0%)

**Table 3:** IgM, ELFA, and IgG avidity results in pregnant women positive for the ELISA detection of IgM anti-CMV antibodies (2005–2007).

Anti-CMV Antibodies				
IgM ELFA		IgG avidity		
Result	No.	High	Moderate	Low
Positive or borderline	19	6 (31.6%)	5 (26.3%)	8 (42.1%)
Negative	9	9 (100%)	0 (0%)	0 (0%)
Total	28	15 (53.6%)	5 (17.9%)	8 (28.6%)

**Table 4:** IgM, ELFA, and IgG avidity in pregnant women by trimester of pregnancy.

Trimester	Anti-CMV IgM		Anti-CMV IgG avidity		
	ELISA	ELFA	High	Moderate	Low
I	25	17	6	4	7
II	2	1	0	1*	0
III	1	1	0	0	1*
Total	28	19	6	5	8

*Seroconversion
